# 764 Burn Advanced Practice Provider Fluorescein Dye Staining Reduces Ophthalmology Consults

**DOI:** 10.1093/jbcr/irae036.306

**Published:** 2024-04-17

**Authors:** Kate E Laramie, Scott W Mueller, Arek J Wiktor, Cameron Gibson

**Affiliations:** University of Colorado Hospital, Denver, CO; UCHealth and University of Colorado School of Pharmacy, Aurora, CO; University of Colorado Hospital, Aurora, CO; University of Colorado, Aurora, CO; University of Colorado Hospital, Denver, CO; UCHealth and University of Colorado School of Pharmacy, Aurora, CO; University of Colorado Hospital, Aurora, CO; University of Colorado, Aurora, CO; University of Colorado Hospital, Denver, CO; UCHealth and University of Colorado School of Pharmacy, Aurora, CO; University of Colorado Hospital, Aurora, CO; University of Colorado, Aurora, CO; University of Colorado Hospital, Denver, CO; UCHealth and University of Colorado School of Pharmacy, Aurora, CO; University of Colorado Hospital, Aurora, CO; University of Colorado, Aurora, CO

## Abstract

**Introduction:**

Facial burn injury patients typically receive ophthalmology consultations irrespective of the severity of their peri-ocular or ocular involvement. Minor facial burns without significant eye involvement or ocular complaints often do not require in-patient ophthalmologist assessment. We sought to assess ocular fluorescein dye staining by burn unit Advanced Practice Providers (APP; Physician Assistants and Nurse Practitioners) to decrease unnecessary ophthalmology consults.

**Methods:**

A prospective evaluation was performed at our ABA-verified burn center for all facial burn injury patients admitted between January 1 through August 31, 2023. Patients who presented with full-thickness injuries to their eyelids or peri-ocular area, those exhibiting tense eyelids raising concern for ocular compartment syndrome, those with severe eye pain and anyone with acute vision loss were excluded from staining and received an immediate ophthalmology consult. For patients with facial burns who did not meet the aforementioned criteria, fluorescein dye stains were administered upon admission by APPs, if available. Ophthalmology consults were only ordered for patients with positive stains.

**Results:**

83 patients were admitted with facial burns between January 1 and August 31, 2023. Of these, 42 patients received fluorescein dye stains by APPs. Among those stained, 79% were male and 21% female. The average age was 49 years old (range 19 to 82). The average TBSA affected was 8.46% (range 0.1% to 38.5%). The majority of patients (83%) had flame/flash burns, 7% had scald burns, 2% had chemical burns, 2% had sun burns and the remainder had unclear etiology. 34 patient dye stains (80%) yielded negative results, obviating the need for an ophthalmology consult. The remaining 8 had positive results, necessitating a consult. Among these, 4 were false positives with no ocular injury found by ophthalmology. The remaining 4 exhibited ocular chemosis, trace punctate epithelial erosions, thermal keratopathy and chronic central epithelial defects from prior Lasik surgery. Ophthalmology was consulted in 37 out of 83 patients, with only 8 consults resulting from APP staining. This signifies a 48% overall reduction in consults placed during this 8-month time frame, and an 80% reduction of consults in whom qualified for the pathway.

**Conclusions:**

Fluorescein dye staining by burn unit APPs minimizes unnecessary ophthalmology consults, likely reducing healthcare costs for patients and healthcare facilities.

**Applicability of Research to Practice:**

Burn centers should consider fluorescein dye stain screening as part of routine practice for burn patients.

